# The right to science and the SDGs: what can we learn from COVID-19?

**DOI:** 10.3389/fsoc.2023.1282721

**Published:** 2023-11-02

**Authors:** Nathaniel T. Weisenberg

**Affiliations:** Center for Scientific Responsibility and Justice, American Association for the Advancement of Science (AAAS), Washington, DC, United States

**Keywords:** human right to science, Sustainable Development Goals, COVID-19, citizen science, science diplomacy, access, participation

## 1. Introduction

In the depths of the COVID-19 pandemic, the astonishing contributions of science and scientists provided reason for hope. The tragic fate of Dr. Li Wenliang, the ophthalmologist who was accused of “spreading rumors” by Chinese authorities after he sought to warn others of the outbreak of the virus, and later died of COVID-19, remains a touchstone in China and beyond (Green, 2020; Xiao et al., 2022). Scientists around the world worked tirelessly to develop vaccines less than a year after the World Health Organization (2020) declared COVID-19 a pandemic (Park and Ducharme, 2021). Many took on additional responsibilities related to public education and countering misinformation, often at significant personal risk (Author, 2021a; Makri, 2021; Nogrady, 2021).

In the United States, Dr. Kizzmekia Corbett, who worked to engage marginalized communities around the importance of COVID-19 vaccination, is credited with saving thousands of lives (Jilani, 2022). In Thailand, Ramida Juengpaisal led the development of a national COVID-19 tracker that provided credible information about the virus and the costs and availability of testing (UN Women, 2020). Thanks in part to the contributions of the scientific community, countries like Canada revamped their approaches to science, technology, and innovation to better align with the best available scientific evidence (Schneegans et al., 2021).

Scientists also published and shared an enormous amount of information on COVID-19, much of it freely available (United Nations Economic and Social Council, 2021). As of August 2023, the Dimensions database records more than 2 million publications related to COVID-19; the WHO COVID-19 Research Database (paused in June 2023) 874,818 results; and the COVID-19 Data Portal more than a million results (United Nations Educational Scientific and Cultural Organization, 2022; COVID-19 Data Portal, 2023; Dimensions, 2023; World Health Organization COVID-19 Research Database, 2023).

This extraordinary amount of activity shows the power of science and scientists in a crisis. In the words of Sianko et al. (2021), “Science never mattered more” (61).

As the 2030 deadline for achieving the United Nations Sustainable Development Goals (SDGs) approaches, the world faces additional interlocking crises, including the “triple planetary crisis” of climate change, biodiversity loss, and pollution (United Nations Climate Change, 2022). These challenges, much like COVID-19, are an opportunity to harness the energy and skills of the global scientific community. The SDGs, as an agreed-upon global framework for sustainable development, and the human right to science, which helps identify priorities, provide a roadmap forward for States to utilize science to address these common challenges with the active involvement of the scientific community.

Doing so will not be easy. Despite some optimism that COVID-19 would spur countries to take stronger actions to realize the SDGs (Ottersen and Engebretsen, 2020; Mohammed, 2021), the pandemic has caused serious setbacks, from the closure of schools to continuing impacts on healthcare systems, that will reverberate for years to come. Efforts to achieve the Goals and the 2030 Agenda for Sustainable Development remain in “grave jeopardy” (2022 SDG Report, p. 3). At the same time, the pandemic demonstrates how in a crisis, societies can indeed act quickly to make systemic change. With the right priorities and points of leverage – utilizing the opportunities and actions called for by the right to science – we can truly “leave no one behind.”

## 2. Relevant subsections

### 2.1. The SDGs, science, and human rights

First, it is worth underlining how the SDGs, science, and the right to science are connected. Both science and human rights are necessary to achieving the SDGs. Goals such as SDG 3 (good health and well-being), SDG 4 (quality education), and SDG 8 (decent work and economic growth) may appear more directly connected to science than others, but all of the SDGs fundamentally depend on the collection and use of accurate data; the building of scientific and research infrastructure, including public funding for research and development; the freedom for scientists to collaborate internationally; the translation and communication of science for the public; and the transformation of scientific research into applications that can be enjoyed by all. Science, technology, and innovation are specifically mentioned in relation to some two dozen SDG targets, but “science cuts across virtually all of the 17 SDGs, as well as their targets and means of implementation; it encompasses such areas as national investment in science, technology, innovation, the promotion of basic science and science education and literacy” (Al-Nashif, 2019).

Just as science underpins the SDGs, so do human rights, without which achievement of the SDGs may benefit some individuals and groups at the risk of perpetuating the marginalization of others. Goals such as SDGs 5 (gender equality) and 16 (peace, justice and strong institutions) directly and evidently connect to human rights, but as the Office of the United Nations High Commissioner for Human Rights (OHCHR) observes, all SDGs are connected to multiple human rights as defined in international human rights instruments [Office of the United Nations High Commissioner for Human Rights (OHCHR), (n.d.)]. As Jensen (2019) describes, “Over 90% of the 169 SDG targets are linked to standards from the international human rights and labor rights frameworks.” Karima Bennoune, the former United Nations Special Rapporteur in the field of cultural rights, observes, “progress on human rights obligations and on the Goals are two sides of the same coin” (Bennoune, 2021; para. 68). Through its emphasis on public access to science and participation in the scientific process, especially through creating opportunities for marginalized groups (Shaheed, 2012; Orellana, 2021; Swiss Commission for UNESCO, 2022; United Nations, 2022), the right to science, specifically, provides a roadmap for advancing scientific progress and sustainable development.

This human rights emphasis is also critical to ensuring that scientific progress is carried out ethically and responsibly, and that everyone benefits from scientific progress. The ways science can be misused to violate human rights and inflict harm are well known [AAAS DoSER (Dialogue on Science, Ethics, and Religion), 2023]. Whether there is a right to also be protected from adverse effects of science and its applications is a topic that deserves more scrutiny (Mazibrada, 2022). Within the scientific community, there is also an increasing focus on scientific responsibility, which the AAAS called in a 2017 statement “inextricably linked to” scientific freedom. “Scientific responsibility is the duty to conduct and apply science with integrity, in the interest of humanity, in a spirit of stewardship for the environment, and with respect for human rights” [American Association for the Advancement of Science (AAAS), 2017]. As Larsen and Pamintuan (2022) have identified, the right to science specifically can be found within multiple SDGs, including 9 (industry, innovation and infrastructure), 12 (responsible consumption and production), and 17 (partnerships for the Goals). Specific targets and indicators, such as internet access (Indicator 9.c.1; Indicator 17.8.1) can serve as ways to both monitor States' progress toward the SDGs and attainment of their obligations under the right to science. Indeed, both the SDGs and the right to science share a fundamental insight: the problems we face are interconnected and cannot be dealt with in isolation.

## 3. Discussion

Here are three recommendations for States that build on the connections between the SDGs and the right to science, along with examples of how the scientific community is currently involved in these efforts.

### 3.1. Take policy actions that address multiple, interlocking issues, building on the interconnectedness of the SDGs

One way to reduce the chance of another global pandemic having such disastrous effects is to address the 17 SDGs together, treating them like an interconnected web. United Nations reports and other research highlight many of these connections, including the linkages among education, sustainable energy, and health, and recommend that States expand educational opportunities for their citizens alongside programs that support income support, job creation, increased health care, social protection, and energy access (Vladimirova and Le Blanc, 2015; Scientific Technological Community Major Group Position Paper for the 2021 High-level Political Forum, 2021).

One example of a policy that takes advantage of these intersections is investing in universal, affordable internet access, mentioned above as an indicator for SDGs 9 and 17. Access to the internet can support even seemingly unrelated SDGs such as 15 (life on land), specifically for land management, land use, and biodiversity conservation (Ekins et al., 2019). The internet also facilitates access to scientific knowledge, which, to be consistent with the right to science, must involve meaningful access to scientific information in ways it can be consumed and used, across a wide variety of platforms.

During the worst of the pandemic, as access to the internet became essential for work, education, and basic services, many countries made urgent efforts to improve internet access, leading to a surge in usage worldwide (United Nations Committee on Economic Social Cultural Rights, 2020, p. 61). However, wide gaps remain between developed and developing countries. Approximately 63% of the global population uses the internet, but only 27% of people in least developed countries do (United Nations Committee on Economic Social Cultural Rights, 2020, p. 61). Closing this gap is important not just for fairness, but will also help advance multiple SDGs and the right to science.

As the COVID-19 examples above show, despite the stereotype of scientists being aloof or cloistered in an ivory tower, there is much evidence of scientists' capacity and interest in taking on urgent problems and in their societal responsibilities (Wyndham et al., 2021). This is no different when it comes to the SDGs. Leading scientific organizations like the American Chemical Society (n.d.) have made using their disciplinary expertise to advance the SDGs a priority, while scientists from lower-income countries have led the way in conducting research aligned with the SDGs (Author, 2021b).

### 3.2. Promote public access to, and participation in, science

Progress toward this key component of the right to science is precisely aligned with progress toward the SDGs. The right to science, including the right to access scientific knowledge, is tied to the human right to information (Kaye, 2020). Adopting policies that support access to information will advance SDG 16 and also have an impact on other SDG targets. Specific measures should include investing in access to the internet, digital and news literacy initiatives, and encouraging meaningful access to publicly held scientific knowledge and participatory (often called “citizen”) science initiatives that engage and include publics, especially marginalized and disadvantaged peoples. States can also encourage science communication strategies that use effective ways of transmitting knowledge, rather than the debunked “deficit model” (Nisbet and Scheufele, 2009; Dudo and Besley, 2016; Reincke et al., 2020).

Participatory science is still an emerging area of scientific practice but has been demonstrated to have multiple benefits, including to improve science literacy (Bonney et al., 2016; Ferreira et al., 2019). During the pandemic, public excitement about science has helped contribute to significant advances in the sectors of epidemiology (Carnegie Mellon University Delphi Group, 2022), clinical care, vaccinations, and treatment, among others. Community efforts can also address areas of the SDGs where governments lack the resources or capacity to collect data (Fritz et al., 2019), including for SDG 3 and SDG 6 (clean water and sanitation) (Fraisl et al., 2020).

Providing opportunities for marginalized groups is also essential. Data collected as part of SDG reporting (especially SDG 4, quality education) can strengthen the case for programs that address educational equity and access to education for women and girls, persons with disabilities, LGBTQIA+ (lesbian, gay, bisexual, transgender, queer, intersex, asexual+) populations, Indigenous communities, people living in poverty and other groups. This is also a key area of focus for the scientific community. In recent years, numerous leading scientific organizations have rededicated their efforts to supporting diversity, equity, inclusion, and accessibility [American Association for the Advancement of Science (AAAS), 2023].

### 3.3. Take strategic actions to strengthen international scientific cooperation and the international science-policy interface

The pandemic has highlighted the life-and-death implications of the connections between science and policy. Science policy and international cooperation have immediate implications for response to pandemics, climate change, and other crises that span borders.

One model highlighted by Colglazier (2020) is the work of the United Nations Technology Facilitation Mechanism (TFM), designed to support countries in achieving the science, technology and innovation goals spelled out by the SDGs, including advancing States' capacities to better address the SDGs in the context of problems brought on by COVID-19. Examples include pilot programs in Ethiopia, Ghana, India, Kenya, Serbia and Ukraine designed to enhance the role of science, technology and innovation in efforts to achieve the SDGs. These efforts were later joined by the European Commission and Japan [United Nations Department of Economic and Social Affairs (DESA), 2022]. International structures such as the TFM that support scientific cooperation and sharing, and other structures that encourage integrating traditional knowledge with international science collaborations, can be especially important for providing aid to disadvantaged populations and involving the input and expertise of the scientific community and other communities.

Cooperation toward achieving the science-related SDG targets will also be strengthened by engagement in science diplomacy. In addition to formal diplomacy, e.g., within multilateral and bilateral agreements, “Track II” diplomacy on a scientist-to-scientist or organization-to-organization level may be particularly effective for sustainable development efforts as it can be less subject to political pressures and tensions between governments. The dialogues between the U.S. and Soviet scientific communities during periods of Cold War tensions are an instructive historical example (National Research Council, 2004; Hecker, 2016).

## 4. Conclusion

The impacts of the pandemic – millions of lives lost, disruptions to education, health care, and much more – have shown the fundamental interconnectedness of our world. A crisis such as COVID-19 ultimately affects us all, and reveals the vast inequalities in current global power structures. At the same time, the transformative capabilities of human knowledge, of science and scientists, provides hope. The SDGs and the right to science, which recognize this interconnectedness and focus on the most marginalized, offer a path forward that specific policy actions, public access and participation in science, and international scientific cooperation can help implement.

Science and scientists are far from perfect. Many different forms of knowledge, and many different communities, have and will need to be involved if the world is to achieve the SDGs by 2030 and if everyone is able to enjoy the benefits and applications of science. Yet, if COVID-19 has taught us anything, it is the transformative potential of a crisis, and the contributions that science can make to addressing enormous global challenges. The right to science provides a lens for not just realizing the SDGs, but for meeting current and future challenges with appropriate urgency.

## Author contributions

NW: Conceptualization, Writing—original draft.
